# The Potential of Sweetpotato as a Functional Food in Sub-Saharan Africa and Its Implications for Health: A Review

**DOI:** 10.3390/molecules26102971

**Published:** 2021-05-17

**Authors:** Flora C. Amagloh, Benard Yada, Gaston A. Tumuhimbise, Francis K. Amagloh, Archileo N. Kaaya

**Affiliations:** 1 Department of Food Technology and Nutrition, School of Food Technology, Nutrition and Bio-Engineering, College of Agricultural and Environmental Sciences, Makerere University, Kampala P.O. Box 7062, Uganda; ampston23@gmail.com (G.A.T.); kaaya.archileo48@gmail.com (A.N.K.); 2 CSIR–Savanna Agricultural Research Institute, Tamale P.O. Box TL 52, Ghana; 3 Root Crops Program, National Crops Resources Research Institute, National Agricultural Research Organization, Kampala P.O. Box 7084, Uganda; yadabenard21@gmail.com; 4 Department of Food Science and Technology, Faculty of Agriculture, Food and Consumer Sciences, University for Development Studies, Tamale P.O. Box TL 1882, Ghana; fkamagloh@uds.edu.gh

**Keywords:** sweetpotato, functional food, plant bioactive compounds, phytochemicals, noncommunicable diseases, type 2 diabetes, sub-Saharan Africa

## Abstract

Increasing urbanization in developing countries has resulted in busier lifestyles, accompanied by consumption of fast foods. The consequence is an increased prevalence in noncommunicable diseases (NCDs). Food-based approaches would be cheaper and more sustainable in reducing these NCDs compared to drugs, which may have side effects. Studies have suggested that consuming functional foods could potentially lower NCD risks. Sweetpotato is regarded as a functional food because it contains bioactive compounds. Recently, sweetpotato has gained attention in sub-Saharan Africa (SSA), but research has focused on its use in alleviating micronutrient deficiencies such as vitamin A deficiency, particularly the orange-fleshed variety of sweetpotato. Some studies conducted in other parts of the world have investigated sweetpotato as a functional food. There is a need to characterize the sweetpotato varieties in SSA and determine how processing affects their bioactive components. This review highlights some of the studies conducted in various parts of the world on the functionality of sweetpotato, its bioactive compounds, and how these are influenced by processing. In addition, the potential health benefits imparted by sweetpotato are expounded. The knowledge gaps that remain in these studies are also addressed, focusing on how they can direct sweetpotato research in SSA.

## 1. Introduction

**Figure 1:**
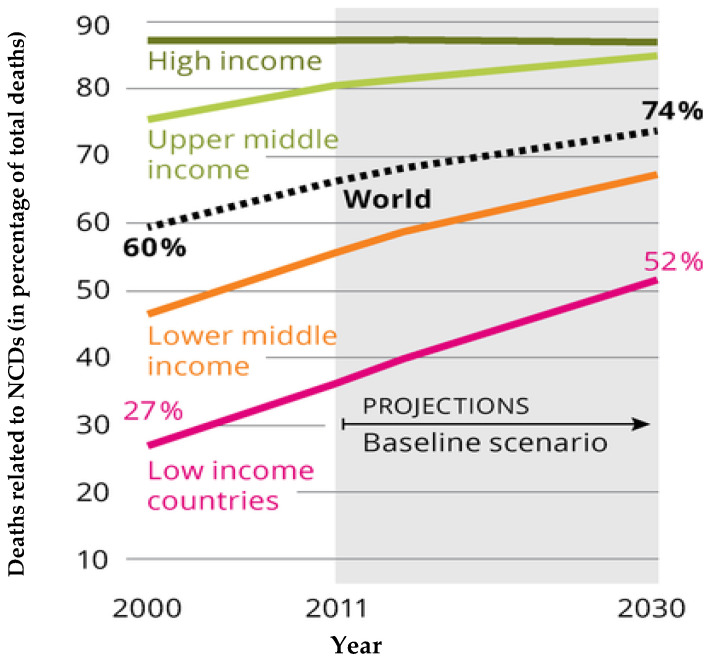
Future development of NCDs across world income regions. Source: European Environment Agency (2017). Downloaded from: https://www.eea.europa.eu/data-and-maps/figures/the-shift-in-global-disease [6].

Noncommunicable diseases (NCDs), especially in developing countries are on the increase [1] (Figure 1). In the last couple of decades, consumers worldwide are becoming increasingly aware of the importance of consuming meals that prevent diseases and promote health [2,3]. Undernutrition and infections are believed to decline with economic development and increased incomes. However, there are attendant changes in diet and lifestyles that have resulted in a shift from consumption of traditional foods to highly processed foods, sugar, and unhealthy fats, as well as lower intake of complex carbohydrates [1,4]. This is the situation in most developing countries [1]. These dietary changes are associated with greater prevalence of obesity and hypertension in the population. The consequence of this is an increased risk of NCDs such as stroke and cardiovascular diseases, inflammatory conditions, metabolic syndrome and diabetes, chronic respiratory diseases, chronic kidney diseases, and cancer, among others [5].

In the light of the current global pandemic (COVID-19), the World Health Organization (WHO) has emphasized that people with NCDs are “among the most likely to become severely ill and die” from COVID-19 [7]. An optimal immune function that can prevent infections such as COVID-19 is dependent on, among other factors, adequate diet and proper nutrition [8]. Generally, an individual’s nutrition status, including consumption of functional foods, are known to promote proper functioning of the immune system [9].

The economic impact of the burden of NCDs is evident in increased personal and national healthcare costs, income losses, decreased productivity, and decreased life expectancy [5]. According to the WHO, in 2018, 71% of global deaths were due to NCDs [10]. It was reported that these NCDs had disproportionately higher rates in low- and middle-income countries, where over 85% of global “premature” deaths (deaths in population aged 30–69 years) due to NCDs occurred [10]. This situation, which poses a serious public health threat to developing countries [1], calls for attention.

Type 2 diabetes mellitus (T2DM), a chronic metabolic disorder, currently affects approximately 422 million people worldwide, with the majority living in low- and middle-income countries [11]. With T2DM having obesity as a highly probable risk factor [12], it is one of the NCDs with an alarming increasing prevalence, especially in developing countries, due to the increased rates of obesity. Between 2013 and 2035, the Africa region, for example, is expected to have as high as a 109.1% increase in the number of T2DM cases [13].

In the past, public health interventions in SSA have focused on communicable diseases and maternal, neonatal, and nutritional disorders. However, NCDs in the region are a growing concern and are now key causes of morbidity and mortality [14]. Among the common, modifiable risk factors that underlie the major NCDs include unhealthy diet [1,10].

Instead of relying on pharmaceutical drugs, with their high costs and associated side effects, to manage the increasing NCD menace, food-based approaches would be a more practical and sustainable solution. Thus, dietary diversity and the regular consumption of cheap and readily available functional foods in SSA such as sweetpotato (*Ipomoea batatas* (L.) Lam, Convolvulaceae) could be encouraged. This could contribute to reducing the incidences of nutrition-related NCDs such as T2DM. Hence, research efforts that focus on these areas are a necessary step in all affected countries.

Sweetpotato, a starchy root crop, can be referred to as a “3-in-1” product, due to its integration of the qualities of cereals (high starch), fruits (high vitamin and pectin content), and vegetables (high vitamin and mineral content) [15]. Sweetpotato roots contain macronutrients such as starch, dietary fiber, and protein, in addition to a broad range of micronutrients including manganese, copper, potassium, iron, vitamin B complex, vitamin C, vitamin E, and provitamin A (as carotenoids, mostly in yellow and orange-fleshed varieties) [16,17,18]. The skin is usually brown, beige, red, or purple, while the flesh color may be white, cream, yellow, orange, or purple [19,20].

Globally, sweetpotato is the seventh most important staple, and in developing countries it ranks fifth, after rice, wheat, maize, and cassava [19]. Among the root and tuber crops cultivated globally, sweetpotato is the second after cassava [18]. As of 2019, the top four global producers of sweetpotato, ranking after China, were all SSA countries: Malawi, Nigeria, Tanzania, and Uganda [21]. Sweetpotato is drought-tolerant once established. It therefore has the potential of improving food and nutrition security, in the mostly rain-fed agriculture in the developing world, where droughts could severely affect yields of other staples such as cereals [22]. It was estimated that more than 2 billion people in Africa, Asia, and Latin America would depend on sweetpotato for food by 2020 [23]. In Uganda, for example, sweetpotato is the fourth most important staple and is grown by over 44% of farmers [24]. Further, it was estimated that by 2018, the biofortified orange-fleshed sweetpotato (OFSP) would have been adopted by over 292,000 Ugandan farming households who would be planting and eating it [24].

Sweetpotato roots are also regarded as a functional food, as they provide, in addition to nutrients, other physiological benefits [20]. They are rich sources of phytochemical compounds such as carotenoids, tocopherols, phenolic compounds, tannins, flavonoids, saponins, and anthocyanins, with their levels varying based on flesh color and variety [20,25]. These bioactive phytochemicals, either singly or collectively, exhibit antioxidant, cardioprotective, antidiabetic, hepatoprotective, neuroprotective, anti-inflammatory, and antimicrobial activities, as well as bowel-regulation properties [26]. The resulting effects are disease-fighting and immune-system-boosting, which ultimately promote health and longevity [27]. The bioactive phytochemicals found in sweetpotato act as potential sources of antioxidants that can scavenge free radicals, and reduce or inhibit cellular damage and reduce metabolic oxidative stress, resulting in disease prevention and better health [17,26].

In recent years, biofortification programs carried out by several countries in SSA, such as Uganda, Malawi, Ghana, Mozambique, Kenya, and Ethiopia, have contributed to the release of new yellow, orange, and purple-fleshed sweetpotato varieties, but mainly OFSP for its provitamin A content [28,29,30,31]. In addition, these sweetpotato varieties may have other optimized traits such as enhanced disease tolerance and early maturity [29,32]. However, the great attention received by the biofortified sweetpotato has primarily been for the purpose of improving nutrition of low-income groups and vulnerable populations, such as children under five and women of child-bearing age [31,33,34].

OFSP, for example, has been highlighted as a choice crop for addressing vitamin A deficiency (VAD) due to its high level of carotenoids, especially β-carotene, the precursor of vitamin A [35,36,37,38]. OFSP has therefore been used in product formulations like complementary foods, crisps, and bread [39,40,41,42]. Generally, sweetpotato has great value in the food industry and has been used for baked foods, confectionaries, and beverages, among other uses [18,43].

Owing to its significant levels of bioactive phytochemicals, it is prudent for research focus on sweetpotato varieties in SSA to shift toward their potential use as functional food and how different processing methods affect the retention of the phytochemicals. In other parts of the world, research has investigated the potential of sweetpotato as functional food; however, such studies are scanty in SSA. Two recent studies in SSA investigated sweetpotato varieties for phytochemicals. The first compared inherent phytochemicals in leaves and storage roots of seven OFSP varieties from Kenya [44]. A second study followed up that evaluated the effect of boiling and frying on retention of some phytochemicals in Kenyan OFSP roots, as well as products from the roots [45]. However, more research is needed to compare not only OFSP varieties, but also other flesh colors. In addition, a broader range of cooking methods that are traditionally applied to sweetpotato in SSA before consumption could be evaluated. This would provide more information on how those methods affect phytochemical retention, and therefore offer recommendations to stakeholders such as farmers, processors, and consumers.

The aim of this review is to highlight recent research conducted mostly in non-SSA countries on the potential of sweetpotato as a functional food, as well as the effect of cooking methods on the availability of the bioactive phytochemicals present. In addition, the review will identify the gaps in knowledge that remain to be addressed, while examining how such research can be applied to sweetpotato varieties in SSA, especially Uganda. Database searches were carried out in Google Scholar, PubMed, and Science Direct for studies relating bioactive compounds in sweetpotato to its functionality. References dating earlier than the year 2000 were not considered.

## 2. Sweetpotato Varieties, Their Distinctive Flesh Colors, and Levels of Bioactive Compounds

There are many varieties of sweetpotato known and cultivated around the world. These varieties come in different storage root skin and flesh colors, shapes, and sizes, and vary in taste and texture. The different varieties of sweetpotato are generally characterized by the skin and flesh color of the storage roots, as well as other agronomic traits such as leaf and stem morphology [46].

Recent research studies have supported the fact that the different varieties of sweetpotato contain different levels of bioactive phytochemical compounds, depending on genetic and environmental factors [47,48,49,50]. The major phytochemicals that are generally present in sweetpotato are flavonoids, terpenoids, tannins, saponins, glycosides, alkaloids, carotenoids, steroids, and phenolic compounds [20,48]. These constituents may vary with varieties depending on flesh and skin color [51,52]. The staple root types in SSA, which are white- or cream-fleshed, are characterized by their high starch content [53]. Other flesh colors range from yellow to pale orange, deep orange, red, and purple. The orange-fleshed ones predominantly contain α-carotene, β-carotene, and β-5 cryptoxanthin [54]. They are usually characterized by their high β-carotene content, with a direct correlation between the intensity of the orange color and level of β-carotene [54].

Purple-fleshed sweetpotato (PFSP) contains higher levels of anthocyanins than other varieties [55]. The antioxidant activities of sweetpotato have mostly been attributed to their phenolic compounds, anthocyanin, and carotenoid contents [49,56]. Phenolic acids such as chlorogenic, isochlorogenic, caffeic, cinammic, and hydroxycinammic, generally present in all sweetpotato varieties, are also associated with their sensory qualities [57,58]. They are more abundant in PFSP and white-fleshed sweetpotato (WFSP) than in the other colored varieties [59].

Phytochemical screening of sweetpotato showed high percentages of reducing sugars and phenolic compounds in WFSP, while OFSP varieties contained higher levels of carotenoids, flavonoids, and total protein [50]. Another evaluation of the phytochemical diversity in sweetpotato roots of different flesh colors (orange, purple, and white) reported that carotenoid levels in OFSP were considerably higher, with β-carotene being predominant. In addition, phenolic acids and flavonoids were higher in PFSP compared to OFSP and WFSP [48].

In addition to variations in flesh color, another study suggested different genes were at work in the flesh versus skin of the sweetpotato, producing various concentrations of phytochemicals and antioxidants. A stronger antioxidant activity was reported in the peels of white and purple varieties when compared to the flesh samples [60]. This demonstrates that the skin of sweetpotato roots is also a rich source of antioxidative phytochemicals. Following this finding, more research is needed to establish if any significant differences exist between peeled and unpeeled sweetpotato roots that have undergone similar processing methods.

## 3. Sweetpotato Bioactive Compounds and Their Potential Health Benefits

Apart from sweetpotato roots being used as a staple food, earlier studies have shown that phytochemicals present in both the leaves and roots may be able to lower the potential health risks posed by free radicals [16,17,55,59]. Table 1 provides a summary of the various health benefits associated with consumption of sweetpotato and the major bioactive compounds responsible for imparting those benefits.

A red-fleshed sweetpotato cultivar grown in the Andean region, for example, has been reported to have higher antioxidant activity and phenolic content than a cultivar of blueberry, a fruit that is widely known to have high levels of antioxidants [61]. Carotenoids, mostly present in OFSP, also have potential antioxidant properties. In a study on OFSP varieties grown in Bangladesh, it was concluded that those varieties could serve a dual role of preventing vitamin A deficiency and providing a source of dietary antioxidants [54]. The relatively high anthocyanins and phenolic compounds in PFSP compared with other flesh colors, as stated earlier, possess antioxidant activities, and play a strong role in the prevention of degenerative illnesses such as cancer and cardiovascular diseases [46,62,63]. Studies have shown that PFSP has preventive properties against colorectal, breast, bladder, and pancreatic cancers [64,65,66,67], as well as elevated blood pressure [68]. Some of the bioactive compounds in sweetpotato and their potential health benefits are discussed below.

### 3.1. Phenolic Compounds

Phenolic compounds occur in tea, coffee, fruits, vegetables, and grains, with sweetpotato also having high levels [57,69]. Phenolic compounds comprise polyphenols, tannins, phenolic acids (e.g., chlorogenic, caffeic, and caffeoylquinic acids), flavonoids, stilbenes, and lignans [70]. Phenolic acids, polyphenols, and flavonoids are the most common phenolic compounds occurring in sweetpotato [71]. Generally, various foods contain complex mixtures of phenolic compounds, although some are specific to particular foods [72].

Since the phenolic compounds found in foods exhibit strong antioxidant activity that could potentially scavenge free radicals, they were thought to primarily curb oxidative damage and protect against lipid peroxidation [73]. However, research conducted in recent times has shown that these compounds may have more complex modes of action than originally thought, and may exert several other biological and physiological effects [72,73].

Epidemiological studies suggest that consuming polyphenol-rich foods reduces the incidence of many chronic diseases; and recent interest in the antioxidant effects of polyphenols have prompted research into the beneficial health effects of plant-based foods, including sweetpotato [91,92,93,94]. In a study on senescence-accelerated prone mice, it was observed that caffeoylquinic acids present in PFSP extracts improved their spatial learning and memory, hence imparting neuroprotection [76]. Another study also reported that chlorogenic acid, in addition to exerting antimutagenic and anticarcinogenic effects, could also prevent hydroxyl radical formation and scavenge free radicals [95]. Other pharmacological properties potentially exhibited by phenolic compounds include hepatoprotective, antibacterial, and hypoglycemic, and they may inhibit HIV replication and cholesterol uptake and prevent endothelial dysfunctions [20,72].

### 3.2. Anthocyanins

Anthocyanins belong to a class of flavonoids, and although a variety of flavonoids exist in sweetpotato roots (e.g., quercetin, myricetin, kaempferol, and luteolin), anthocyanins are mainly responsible for pale pink to purple colors of the flesh of sweetpotato [51]. PFSP roots typically have higher anthocyanin contents, whereas white-, yellow- or orange-fleshed roots have little or no anthocyanin content [96,97]. PFSP roots, when compared to other highly pigmented vegetables such as red onion, purple asparagus, and eggplant, showed higher antioxidant activity [98]. Many researchers reported that PFSP anthocyanin could scavenge free radicals, enhance memory function, inhibit cancer cell growth, attenuate liver dysfunction, decrease blood sugar, and lower insulin resistance [18].

It has been observed that PFSP extracts showed protective effects to hepatic insulin resistance via blocking oxidative-stress-mediated endoplasmic reticulum stress in the liver of mice that were given a high-fat diet. Additionally, it improved the fasting blood glucose level, glucose, and insulin tolerance by suppressing the production of reactive oxygen species and restoring glutathione content and activity of antioxidant enzymes in mice [79]. Hypoglycemic effects of anthocyanins from PFSP roots were also reported to be associated with the inhibitory effects toward α-amylase and α-glucosidase activity, which may decrease blood glucose levels [80]. Additionally, PFSP extracts could improve metabolic parameters closely related to obesity, decreasing liver injury and attenuating fatty liver disease in mice fed a high-fat diet [85,87].

Treatment of human colonic SW480 cancer cells with anthocyanin extracts from PFSP roots caused a dose-dependent decrease in colonic cell numbers. These results suggest the capacity of anthocyanin-enriched PFSP to protect against colorectal cancer by inducing cell-cycle arrest, antiproliferative, and apoptotic mechanisms [64,74,75].

Anthocyanins have also been shown to have a beneficial effect on the gut microbiome by increasing the populations of good bacteria and decreasing the populations of *Staphylococcus aureus* and *Salmonella typhimurium*. This shows that these anthocyanins might exert prebiotic-like activity through a positive modulation of intestinal microbiota [77].

The animal studies that have been conducted to demonstrate the health-promoting effects of sweetpotato have largely depended on raw sweetpotato extracts. Though these extracts have shown potential, consumers eat sweetpotato processed as food, not in extract form. Therefore, questions arising from such research include how much of the bioactive compounds are present and bioavailable in the cooked sweetpotato, and how much is required to elicit a favorable response. Because the food matrix will influence how the gastrointestinal tract processes nutrients, it would therefore be prudent to generate data on the functional potential of cooked sweetpotato roots.

### 3.3. Carotenoids

Carotenoids are yellow, orange, or red pigments that widely exist in fruits and vegetables having those colors (carrots, tomatoes, papaya, sweetpotatoes, etc.), but also in green leafy vegetables such as lettuce, spinach, and kale, where chlorophylls conceal the color of these compounds [99]. To date, more than 750 different carotenoids are known, but only about 40 are consumed in significant amounts in the diet of humans. Of these, α-carotene, β-carotene, β-cryptoxanthin, zeaxanthin, lycopene, and lutein are the most abundant [100].

Carotenoids were originally considered a functional ingredient because of their provitamin A activity (α-carotene, β-carotene, and β-cryptoxanthin). Vitamin A, apart from being a nutrient for visual acuity, is also required by the body to maintain the integrity of healthy mucous membranes and skin [20].

OFSP is one of the best sources of β-carotene, with up to 63-fold more β-carotene than non-OFSPs. β-carotene levels differ among OFSP varieties; for instance, the OFSP varieties released in Uganda over the past decade have β-carotene contents ranging from 3.76–31.45 mg/100 g on a dry-weight basis [28,29,101]. This trait segregates highly in breeding populations, with a high heritability, making it possible for continuous genetic improvement of β-carotene in sweetpotato varieties in SSA [102]. It is noteworthy that the β-carotene present in OFSP can raise serum vitamin A levels [31], and roots of some OFSP varieties, particularly the ones with deep orange flesh color, have the potential to contribute ≥100% of the recommended dietary allowance for vitamin A of 4- to 8-year-old children [31,36,37,38,103].

In addition to their provitamin A activity, carotenoids have been demonstrated to exert health benefits through their action as radical scavengers and protective activity against macular degeneration, cardiovascular diseases, and mutagenesis and tumor formation [20]. There is strong evidence that the regular consumption of carotenoids can reduce the risk for lifestyle-related diseases [38]. This buttresses the need for non-nutritive research (example, functional food properties) to be undertaken with OFSP varieties in the form that is consumed in SSA. A study demonstrated that puree from OFSP roots may have prebiotic potential by increasing the population of *Bifidobacterium* spp., and stimulating the production of short-chain fatty acids, especially butyric acid, a well-known compound favorable to gut health [89].

### 3.4. Dietary Fiber, Resistant Starch, and Effect on Glycemic Index

Dietary fiber is a term encompassing a variety of edible non-starch plant polysaccharides (such as cellulose, hemicellulose, pectin, and lignin) that are resistant to digestion by gastrointestinal enzymes [51,104]. Dietary fiber occurs naturally in a variety of grains, legumes, fruits, and vegetables [104]. Dietary fiber can be classified as either soluble or insoluble fiber. Soluble dietary fiber partially dissolves in water to form a gel-like material. It undergoes fermentation to yield end products that are beneficial to health by improving the colonic environment, modulating glucose and lipids, and regulating immune responses. Examples of soluble fiber are gums, pectin, mucilage, inulin, beta-glucan, and hemicellulose [104,105]. Insoluble fiber, on the other hand, does not dissolve in water. It passively attracts water and imparts a health benefit by increasing bulk and softening stool, thus decreasing the transit time of digested food in the colon and preventing constipation [89]. Insoluble dietary fiber includes lignin, cellulose, modified cellulose, and larger fractions of hemicellulose [104].

In addition, the growth of certain microbes, mostly lactic acid bacteria, is greatly improved by dietary fiber, which serves as food for the microbes (prebiotics) [106]. These beneficial microbes usually grow at the expense of pathogenic microbes, which are subsequently displaced from the colon and expelled [106]. Thus, in general, increased fiber consumption is linked to reduced prevalence of chronic diseases and promotion of health [106]. Some of the mechanisms involved in the health-promoting effects of dietary fiber include antioxidant properties, carcinogen-binding, production of short-chain fatty acids, reduced caloric density of foods, increased excretion of cholesterol [106], better management of blood glucose, improved gut health, and reduced risk of coronary heart disease [104]. Compounds produced as a result of dietary fiber metabolism, especially the short-chain fatty acids, are regarded as anti-inflammatory and could be important in warding off oxidative stress and inflammation before and during acute infection, such as occurs in COVID-19 cases [8].

The dietary fiber content in sweetpotato roots varies depending on the genetic characteristics and growing conditions [52] and may be up to 3.0 g/100 g on a fresh weight basis [20]. Regardless of the variety, sweetpotato is reported to be beneficial to T2DM patients due to its high dietary fiber content and moderate glycemic index (GI). This is because fiber is associated with stabilization of blood sugar and lowering of insulin resistance [107].

Pectin, a heteropolysaccharide, extracted from sweetpotato roots was found to exhibit a good antioxidant activity [108]. Some sweetpotato varieties of Indonesian origin were also found to contain prebiotic components that were positive for the growth of the beneficial bacteria *Lactobacillus plantarum* and *Bifidobacterium longum* [109].

Depending on the variety, sweetpotato starch content varies between 50 and 80% on a dry-weight basis [107]. The most important factors affecting nutritional value of starch include the rate of digestion along the gastrointestinal tract (GIT) and the subsequent metabolism of its monomers [107]. Rapidly digestible starch (RDS) can be hydrolyzed to glucose within 20 min after contact with amylase and other enzymes, leading to a quick elevation of blood glucose. Slowly digestible starch (SDS) is broken down slowly within the small intestine and may be hydrolyzed to glucose within 20–120 min, providing sustained glucose release. Resistant starch (RS) is a starch fraction that is resistant to enzyme digestion and usually passes unchanged and unabsorbed from the stomach into the small intestine [106]. It is usually hydrolyzed after 120 min, being typically fermented by colonic microbiota [107,110]. Resistant starches may be resistant to digestive enzymes because the compact nature of the molecules may not allow the enzymes to reach the starch granules [111]. Resistant starches contribute fewer calories than regular starch molecules during digestion in the GIT [106]. Resistant starch has similar physiological benefits to dietary fiber [111].

High-starch foods are usually considered high GI and have been associated with the development of metabolic disorders such as T2DM [81]. However, sweetpotato starch is considered moderate GI, with an average value of 55.07 compared to 85.46 for potato starch, which is high GI. This varies among locations, varieties, maturities, cooking methods, cooling processes, and storage conditions [107].

The benefits of consuming a diet rich in SDS and RS, such as occurs in sweetpotato [112,113], are as a result of its moderate impact on GI. A low to moderate GI diet is linked to a reduced risk of T2DM and cardiovascular diseases, while a positive correlation has been found to exist between the consumption of high GI foods and an increased risk of chronic NCDs [90]. When hyperglycemic rats were fed sweetpotato starch for 4 weeks, there was an improvement in their postprandial glycemic response compared to their counterparts fed on high GI potato starch [81,82]. The results also indicated that sweetpotato starch could improve insulin sensitivity. Consumption of RS has been associated with improvements in insulin resistance and reduced accumulation of adipose tissue, leading to decreased risk for metabolic diseases [110,114]. RS found in some Sri Lankan sweetpotato varieties was as high as 17.2% [115]. However, available literature on the characterization of resistant starch from sweetpotato is limited [51]. This could be regarded as a prospective area of research for SSA sweetpotato varieties.

## 4. Effects of Postharvest Processing and Cooking on Sweetpotato Bioactive Compounds

Domestic food-processing methods aim to make the final product more flavorful, tastier, more digestible, and microbiologically safer [99]. However, postharvest processing and heat treatments applied to foods, including sweetpotato roots prior to consumption, can cause changes in their chemical composition and impact the levels and bioavailability of their bioactive compounds [99]. Table 2 summarizes the effect of different cooking methods on the retention of sweetpotato bioactive compounds.

### 4.1. Phenolic Compounds

Some of the polyphenols present in sweetpotato are in the bound form (usually bound to fiber), and they are released during cooking [119]. Heat increases the release of phenolic compounds through the hydrolysis of glycosidic bonds, and also inactivates the enzyme polyphenol oxidase, which is responsible for degradation of these compounds in fresh sweetpotato roots [117]. Furthermore, the enzymatic reactions that occur during mechanical processes such as peeling and chopping may explain the various degrees of losses of phenolic compounds [99]. Thus, research into primary processing methods such as peeling is warranted.

It was observed that, compared to other cooking methods such as frying and microwaving, boiling was less friendly for conservation of phenolic acids and flavonoids, and led to as high as 20% losses in phenolic acids [99]. A previous study concluded that steam cooking resulted in statistically nonsignificant increases in the content of phenolic compounds in sweetpotato leaves and roots [116]. An investigation that compared boiling, baking, frying, and microwaving of sweetpotato roots showed that except for boiling, the other cooking methods increased phenolic compounds content. The decrease in phenolic compounds through boiling was attributed to their leaching into water because of their hydrophilic nature [117].

Other researchers who worked on OFSP and PFSP grown in Croatia and Slovakia observed a similar trend where the total phenolic content in all heat-treated samples, except boiling, were higher compared to the raw samples [118]. Losses of up to 39% occurred in boiled samples. The general increase in phenolics in cooked samples was attributed to the release of bound phenolics during cooking [118]. On the contrary, in a study conducted in Brazil, researchers observed an increase in phenolic compounds content after boiling in two of their four biofortified sweetpotato varieties, and a decrease in the other two [119]. These observations confirm the variations in phytochemical constituents of different sweetpotato varieties in different locations, and thus necessitate more research into local Ugandan varieties.

### 4.2. Anthocyanins

Anthocyanins are highly reactive molecules sensitive to degradation reactions. Their stability is influenced by their structure and concentration, the presence of enzymes, oxygen, pH, or temperature [127]. There are mixed findings on anthocyanin content due to processing. Some researchers observed a 34% lower content of anthocyanins in steamed deep PFSP and a 41% decrease in boiled samples compared to raw samples [120]. Similarly, an almost 50% reduction in PFSP anthocyanins after steaming has been reported [121]. Conversely, another study reported an increase in anthocyanins after cooking. There was a 3.7-fold increase when boiled in water, 4.3-fold when steamed, 6.6-fold when baked, and 8.5-fold after microwaving [118]. This phenomenon was attributed to the fact that most anthocyanins are stable at high temperatures because of the formation of more stable oligomeric pigments, which increase with increasing temperature. The second reason was the fact that anthocyanins in sweetpotato occur in acylated forms with various phenolic acids, making the anthocyanins more resistant to heat, pH, and light sensitivity [118]. In another study, it was reported that air-fried, deep-fried and stir-fried cooking resulted in a decrease of total anthocyanins by 45%, 32%, and 31% respectively, while baking decreased it by 11%, boiling increased it by 6.55%, and the results from steaming and microwaving were inconclusive [122].

### 4.3. Carotenoids

Generally, most researchers have observed total carotenoid losses with pretreatment and cooking, while others have reported losses only with specific cooking methods and an increase in concentration with other methods. Carotenoids are highly unsaturated molecules, causing them to be sensitive to light and oxygen, and hence susceptible to degradation [128]. The degradation level depends on the cooking method used, as well as time and temperature conditions. A greater degradation of carotenoids has been reported with higher temperatures and longer processing times [99]. Domestic pretreatments generally applied to vegetables, such as washing, peeling, removal of nonedible parts, and cutting, greatly reduce the final carotenoid content of foods because of the exposure of the inner tissues to light and oxygen [129].

Thermal processing may, however, lead to increased bioavailability of carotenoids due to greater chemical extractability and breakdown of the food matrix [130]. An investigation of the effects of different cooking methods on vegetables reported that sweetpotato retained 86.1% of β-carotene during induction boiling, compared with 75.9% during conventional boiling and 66.4% during microwave steaming [123].

In other research conducted on five sweetpotato varieties, all three cooking methods applied (boiling, steaming, and roasting) resulted in decreased total carotenoid content, although the level of retention in boiled samples was higher compared with steamed and roasted [120]. Similarly, other researchers observed that baking and microwaving decreased carotenoid levels in sweetpotato; however, boiling and frying increased carotenoid concentrations when compared to raw samples [117]. They attributed the increase in carotenoids of fried samples to a greater capacity for β-carotene extraction due to changes in cell-wall structure caused by oil at a high temperature. Further, for the boiled samples, they opined that carotenoids linked to some proteins dissociate in water during boiling, thus increasing their concentration [117].

All traditional processing methods (boiling, steaming, baking, and deep-frying) applied to OFSP in another study generally decreased β-carotene content [124]. For all five OFSP varieties evaluated, the boiled, steamed, and deep-fried samples retained more all-trans-β-carotene (the major provitamin A carotenoid in sweetpotato) compared with baked samples. All-trans-β-carotene is the preferred substrate in β-carotene metabolism compared to its cis-isomers, and it contributes twice as much to the amount of retinol activity equivalents [124]. Similarly, in a study investigating the effect of traditional processing methods and different drying procedures on carotenoid retention in OFSP storage roots, it was reported that the average amount of all-trans-β-carotene decreased with all processing methods [125]. Boiling for 20 min and deep-frying for 10 min each reduced the all-trans-β-carotene by 12%, while steaming for 30 min led to a reduction of 23%. Drying of OFSP slices at 57 °C in a forced-air oven for 10 h reduced this parameter by 12%. Solar drying and open-air sun drying, both to a moisture content of 10% or less, reduced the all-trans-β-carotene by 9% and 16% respectively [125].

### 4.4. Starch

Different factors, including the food sample, starch structure, amylose and amylopectin ratios, processing method, and other components of the food matrix could alter textural and rheological properties of starch in food. These factors could result in differences in starch digestibility and the GI of a particular food [107]. It has been indicated that the kinetics of sweet potato starch digestion and GI are influenced by the variety and the processing methods employed [107].

Raw starch is generally more resistant to enzymatic hydrolysis in the GIT than gelatinized starch, and raw sweetpotato starch, like other tuber starches, behaves in the same way [107]. However, only a small amount of starch is consumed in the raw state, and foods are usually subjected to thermal and/or moisture processing for varying times before consumption. During cooking, plant cell walls are softened through swelling and cell separation, causing the structure of the starch molecules to change and individual molecules to be released [124]. Precooking of food, followed by cooling (such as in refrigerated storage), and then reheating prior to consumption results in the starch becoming resistant to digestion (converted to RS), thereby lowering its glycemic response [90,131].

The effects of baking, steaming, and microwaving (as traditional cooking methods) and dehydrating on the GI of a popular sweetpotato variety, Beauregard, was investigated using 12 human subjects [126]. It was reported that irrespective of the cooking method used, there was no significant difference in the GI, which ranged between 63 and 66. However, the dehydrated sweetpotato had a low GI of 41. The study concluded that sweetpotato could be regarded as a medium GI food, irrespective of the cooking method applied [126]. In another study, when French fries prepared from five different sweetpotato varieties were compared, interestingly, the GI values varied from low (52.16) to moderate (58.08) among the different varieties [112]. As expected, the varieties that showed higher proportions of SDS and RS exhibited lower GI values, compared to the varieties with lower SDS and RS levels. Apart from the higher SDS and RS proportions contributing to the lower GI [112], it was also suggested in a review [107] that the lower GI in the fried samples compared with the moderate GI in the baked, steamed, and microwaved samples from the previous study [126] could be due to the formation of amylose–lipid complexes. These complexes are thought to slow down starch digestion and delay gastric emptying, thereby resulting in lower glycemic response [107].

The processing of Jamaican sweetpotato roots by boiling led to a lower GI in comparison with baking, roasting, and frying [90]. Boiled samples had the lowest GI values of between 41 and 50, fried samples were between 63 and 77, baked samples had GI of 82–94, and roasted samples had GI of 79–93. The conclusion was that the GI of Jamaican sweetpotato roots was influenced greatly by the cooking method, and to a lesser extent, the variety. The lower GI in boiled samples compared with the other methods was attributed to the fact that boiling retained larger amounts of RS [90].

In sweetpotato, the knowledge that processing can modify the nature of its starch, thereby modulating postprandial blood glucose response in consumers [126], is of great significance. Understanding the effect of different cooking methods on sweetpotato GI and glucose availability can provide guidance to consumers as well as processors for the development of functional foods such as sweetpotato-based low-GI foods. Thus, investigation on processing methods using local varieties is vital.

## 5. Bioavailability of Bioactive Compounds in Sweetpotato

The potential health benefits of bioactive compounds are not only dependent on the amounts consumed, but also on their bioavailability; i.e., biological availability [132]. Bioavailability refers to the proportion of a specific ingested nutrient or component in food that becomes available for an organism to utilize in its normal physiological functions [133,134]. Even though a compound has strong antioxidative or other biological activities in vitro, it would have little biological activity in vivo if little or none of the compound reaches the target tissues [132].

Bioavailability involves two other terms: bioaccessibility and bioactivity [134,135]. Bioaccessibility is the fraction of an ingested compound that is released from its food matrix in the GIT and thus becomes available for intestinal absorption [135]. Bioactive compounds are generally absorbed in the small intestine by transport mechanisms such as passive diffusion, carrier-mediated transport, and receptor-mediated transport [136]. Bioaccessibility therefore relates to the digestive transformation of food that can be absorbed by the organism [134]. Bioactivity refers to a specific effect obtained after exposure to a metabolite. It includes tissue uptake and the respective physiological response [137]. In general, bioavailability is influenced by storage conditions of food, processing method, type of bioactive compound being considered, dose ingested, food matrix structure and composition, presence of components that affect absorption efficiency, and host-related factors [138,139,140]. Thermal processing can either increase or decrease the bioaccessibility of a bioactive compound [132].

### 5.1. Carotenoids

The bioavailability of carotenoids has been reported to be between a wide range of 3.5% and 90% [100]. The extent of changes in the profile and content of carotenoids is generally proportional to the temperature and duration of cooking [132]. Heat processing may generally increase the relative bioaccessibility of consumed carotenoids by destruction of cell walls and organelle membranes housing these carotenoids [141]. This results in the release of the carotenoids from the food matrix for the effective action of digestive enzymes [132]. It has also been suggested that heat denatures protein–carotenoid complexes, thereby favoring their release from the food matrix [100].

Generally, heating in the presence of oil and co-consumption of dietary fats and oils with carotenoids also promotes carotenoid bioaccessibility and absorption [132,141]. Among several reasons assigned to this phenomenon is the fact that dietary fat serves as a sink for the solubilization of carotenoids and other lipophiles liberated from the food matrix [141]. Secondly, dietary fat stimulates secretion of lipases and bile salts into the intestine for digestion of lipids and mixed micelles that solubilize carotenoids [141]. In addition, a higher intake of fat in a meal is thought to delay gastric emptying, thereby providing additional time for the lipophilic molecules like carotenoids that are within mixed micelles to be liberated and solubilized [141]. When OFSP flour diets with varying amounts of dietary fat were fed to Mongolian gerbils, it was detected that fat enhanced the bioactivity of β-carotene [142]. This was proved by a higher liver vitamin A storage and a more efficient conversion of β-carotene into vitamin A in the gerbils fed higher fat [142].

The effect of the food matrix on the bioaccessibility of carotenoids has been reported to be dependent on the specific food being considered [141]. Boiling carrot slices, for example, disrupted the cell walls and increased carotenoid bioaccessibility, whereas the same treatment applied to yellow peppers showed no disruption of the cell walls and therefore did not increase carotenoid bioaccessibility [143]. This suggests that the cellular structure and molecular composition of the food matrix influences the bioaccessibility of carotenoids [141].

Prior works on sweetpotato roots have provided evidence that although cooking methods employed prior to consumption may decrease the retention of the total amount of β-carotene, the bioaccessibility may be increased significantly [124]. Heat-processed OFSP had significantly greater bioaccessible β-carotene levels compared with raw samples. When steaming, boiling, baking, and deep-frying were compared, the bioaccessibility of deep-fried samples was highest, followed by steaming/boiling, and then baking [124]. It was observed that the microstructure of steamed and fried sweetpotato roots played an important role in the food matrix softening, disintegration, and subsequent release of β-carotene during in vitro gastric digestion. Frying was reported to cause a disruption of cells and the creation of a porous structure, compared to steaming, which produced a more compact structure. This phenomenon resulted in a quicker release of β-carotene in the fried samples than in the steamed samples during digestion. The low β-carotene bioaccessibility of the baked samples was opined to be because of hardening of the surface of the samples during baking that limited the disruption of the sweetpotato matrix [124].

When the effect of thermal processing and oil addition on β-carotene bioaccessibility in OFSP samples was investigated [144], it was reported that the average amount of all-trans-β-carotene decreased after thermal processing. However, bioaccessibility was enhanced after in vitro digestion. For fresh storage roots, it was observed that initial homogenization of tissue, followed by boiling and the addition of oil, significantly increased the bioaccessibility of all-trans-β-carotene compared with the samples that were initially boiled, followed by pureeing and oil addition [144]. Based on these findings, it was indicated that the extent of cell-wall rupture is the most important determinant of the in vitro bioaccessibility of carotenoids in OFSP. For OFSP flour, when oil was added to the sample before making it into porridge, the result was a higher bioaccessibility of all-trans-β-carotene in favor of the sample that was made into porridge before the addition of oil [144]. The researchers proposed that the presence of fat during the cooking of porridge had a positive effect on the incorporation of all-trans-β-carotene into micelles, thereby enhancing bioaccessibility.

### 5.2. Phenolic Compounds

The most abundant phenolic compounds in the human diet are not necessarily the ones with the best bioavailability [72]. This underscores the importance of evaluating the bioavailability of a nutrient, in addition to knowledge of the amount present in a food sample. On average, 24% of total phenolic compounds in a food matrix occur in the bound form [145]. During food processing and digestion, the bound phenolic compounds are released, after which they tend to be absorbable and metabolized in the GIT [145].

The bioaccessibility of polyphenols is affected by both physical and biochemical changes. These include structure, such as presence of cell-wall material in the food matrix, interaction with other micro- and macromolecules [145], methods of preparation and cooking, the specific food matrix involved, and host-related factors such as an individual’s age and gut microbiome [72]. Thermal processing of food and gastrointestinal digestion have been found to significantly increase the bioaccessibility of phenolics and their antioxidant activity, probably due to the breakdown of polyphenol–protein complexes [71,132].

Although the release of polyphenols generally occurs during the gastric phase of digestion, different phenolic compounds from PFSP were found to be released continuously during the entire digestion process [71]. When comparing polyphenol bioaccessibility in potato with sweetpotato, it was observed that sweetpotato polyphenols increased significantly only after intestinal digestion, unlike potato polyphenols, which were detected after the gastric phase [69]. Most phenolic compounds are assimilated in the small intestine, but some are fermented by the action of microflora in the colon [146]. Polyphenol metabolites formed by the action of colonic microflora could significantly enhance the total phenolic bioavailability [145].

The interaction of phenolics with other compounds has been reported to affect the bioavailability [145]. Dietary fat was reported to enhance the absorption of flavonoids, while dietary fiber appeared to delay the absorption [72,147]. The capacity of polyphenols and their metabolites to bind to proteins may delay their delivery to cells and tissues [148].

## 6. Antidiabetic Potential of Sweetpotato in Sub-Saharan Africa

Although the increasing prevalence of T2DM in developing countries is of concern, not much research has focused on available crops; for example, sweetpotato, present in SSA, and its antidiabetic activities. While in 2013, the African region was estimated to have the lowest prevalence (19.8 million) of adults aged 20–79 years with T2DM, it was projected to have the highest (41.4 million) proportional increase by 2035, an increase of 109.1% [13]. Uganda, for example, was projected to have an increase of 166.9% in the number of adults suffering from T2DM between 2013 and 2035 [13].

Sweetpotato may have potential antidiabetic activity, as has been demonstrated using extracts [80,83,84,149]. While these studies may provide some information on the antidiabetic potential of sweetpotato, much of the research was conducted using sweetpotato extracts, with concentrations much higher than those in the human diet. Since sweetpotato is not consumed in extract form, but usually after cooking whole roots, antidiabetic potential studies relying on the consumed forms, rather than extracts, would be more beneficial to stakeholders.

Many available synthetic drugs widely used in the management of T2DM act by inhibiting α-amylase and α-glucosidase, the enzymes that hydrolyze starch into glucose in the GIT, thereby reducing hyperglycemia [150]. However, these inhibitors are reported to cause several side effects, such as abdominal distention, flatulence, and diarrhea [151]. Therefore, food-based approaches, being natural, may be safer and more tolerable than α-amylase and α-glucosidase inhibitors. Consuming foods with complex carbohydrates and high dietary fiber like sweetpotato roots has been found to delay the rate of carbohydrate conversion to glucose by inhibiting the activities of α-amylase and α-glucosidase [152]. Functional foods in general, sweetpotato as an example [20], containing bioactive compounds such as polyphenols have also shown enhanced antioxidant, anti-inflammatory, anti-cholesterol, and increased insulin-sensitivity functions, resulting in lowering of blood glucose levels in patients with T2DM [153].

## 7. Areas of Future Sweetpotato Research in Sub-Saharan Africa

Sweetpotato, having bioactive phytochemicals as presented in this review, may have potential antidiabetic activity. Studies using extracts showed that sweetpotato exhibited potential antidiabetic activity [80,83,84,149]. However, not much research has focused on antidiabetic activities of sweetpotato varieties bred in SSA. Research on how cooked sweetpotato, the form mainly eaten in SSA, is warranted to find the evidence needed before recommendation to people with diabetes or insulin resistance to help control blood glucose. This diet therapy would be cheaper than conventional drugs and may have fewer side effects.

The growing conditions of sweetpotato are aptly suited for SSA and are therefore inexpensive and readily available. In addition, the transformation of sweetpotato roots into value-added marketable products is increasing [18]. There is therefore the need for characterization of our varieties available for their bioactive components. From the literature, these bioactive compounds have been documented, especially in other parts of the world such as Asia and the United States. However, there is a knowledge gap between the theoretical bioactivity of these compounds and their actual influence on the body, once ingested. There is no extensive research on their bioaccessibility after consumption, especially with respect to the effects of the food matrix and processing changes. Therefore, to fully understand the potential of sweetpotato varieties present in SSA as functional food, research is needed to explore the levels and bioaccessibility of their bioactive compounds, taking into consideration the various preparation and processing methods for maximum retention of these compounds.

## 8. Conclusions

In SSA especially, NCDs and metabolic disorders are steadily increasing, thereby prompting the need to fully understand how food-based approaches complement the current drug-based treatments. Although sweetpotato is an important food globally, it is only in recent years that research on this food crop has focused on its bioactive compounds, and hence its potential as a functional food. This review has shown that sweetpotato contains bioactive compounds such as carotenoids, polyphenols, dietary fiber, and RS. These compounds have been reported to play a role in modulating some metabolic processes, thereby imparting health benefits to humans. This review has further presented evidence on why sweetpotato can be regarded as a functional food and its preventive role against NCDs. However, there remains a gap to be addressed with regard to characterization of SSA sweetpotato varieties, how common processing methods employed by households in SSA affect the retention of their bioactive compounds, and the bioavailability of these compounds. These research efforts will provide holistic information on the functionality of sweetpotato in reducing NCDs among the individuals living in SSA.

## Figures and Tables

**Table 1:** Health benefits associated with sweetpotato consumption.

Health Benefit	Bioactive Compound	Sweetpotato Flesh Color	References
Antioxidant capacity (scavenge free radicals)	Phenolic compounds, anthocyanins, carotenoids, tocopherols, flavonoids, ascorbic acid	White, cream, yellow, orange, purple	[44,55,59,61,62,63]
Anticancer properties (colorectal, bladder, breast, pancreatic, lung, prostate)	Anthocyanins, ascorbic acid, carotenoids	Orange, purple	[64,65,67,74,75]
Neuroprotection	Caffeoylquinic acid, anthocyanins	Purple	[76]
Reduction in systolic blood pressure	Anthocyanins	Purple	[68]
Hepatoprotective (improved liver function)	Anthocyanins, phenolic compounds	White, purple	[18,68]
Antimicrobial	Phenolic compounds, anthocyanins, flavonoids	White, cream, purple	[20,72,77,78]
Antidiabetic (decrease blood sugar and lower insulin resistance)	Phenolic compounds, dietary fiber, resistant starch	White, cream, orange, purple	[79,80,81,82,83,84]
Antiobesity	Anthocyanins, dietary fiber, resistant starch	White, purple	[85,86,87]
Anti-inflammatory	Anthocyanins, carotenoids, phenolic compounds, ascorbic acid	Yellow, orange, purple	[74,88]
Prebiotic and bowel regulation	Anthocyanins, carotenoids, dietary fiber, short-chain fatty acids	Orange, purple	[77,89]
Cardiovascular protection	Carotenoids, dietary fiber	Orange	[20,90]

**Table 2:** Effect of different cooking methods on the retention of sweetpotato bioactive compounds.

Bioactive Compound	Processing Method Applied	Sweetpotato Flesh Color	Effect on Retention	References
Phenolic compounds	Steaming	Orange	There were statistically nonsignificant increases in concentrations of both total phenolics and individual phenolic acids after cooking	[116]
	Boiling, baking, frying, microwaving	Cream	Boiling decreased phenolic compounds concentration, while the other methods increased it	[117]
	Boiling, steaming, baking, microwaving	Orange, purple	Except for boiling, all other cooking methods increased total phenolic content	[118]
	Boiling, steaming, roasting, flour	Orange	Steaming, roasting, and flour processing decreased phenolic compounds, while boiling resulted in decreases in two of four varieties and increases in the other two	[119]
Anthocyanins	Boiling, steaming, baking, microwaving	Purple	All cooking methods increased anthocyanin content, with microwaving being the highest	[118]
	Boiling, steaming, roasting	White, yellow, orange, purple	Anthocyanins were barely detected in white, yellow, and orange types. For the purple, all cooking methods decreased total anthocyanin content	[120]
	Steaming, baking	Purple	Steaming reduced total anthocyanin content by nearly half, while baking decreased it by 19%	[121]
	Boiling, steaming, baking, microwaving, deep frying, air frying, stir frying	Purple	Boiling increased total anthocyanin content, steaming and microwaving had no significant effect, but baking and all frying methods decreased it	[122]
Carotenoids	Boiling, baking, frying, microwaving	Cream	Boiling and frying increased total carotenoid concentrations, while baking and microwaving decreased it	[117]
	Boiling, steaming, roasting, flour	Orange	All methods decreased total carotenoid content, with flour processing exhibiting the greatest degradation	[119]
	Boiling, steaming, roasting	White, yellow, orange, purple	All cooking methods decreased total carotenoid content	[120]
	Induction boiling, conventional boiling, microwave steaming	Not specified	All methods decreased β-carotene content, with microwave steaming decreasing it the most	[123]
	Boiling, steaming, baking, deep frying	Orange	All methods generally decreased β-carotene content, with baking decreasing it the most	[124]
	Boiling, steaming, deep frying, drying (forced air convection, solar, open air)	Orange	All processing methods generally decreased β-carotene content, with solar drying retaining the most and steaming retaining the least	[125]
Starch	Boiling, baking, frying, roasting	Not specified	The GI increased in the order boiling < frying < roasting < baking	[90]
	Frying	Not specified	All fried samples had low to moderate GI	[112]
	Steaming, baking, microwaving, dehydrating	Orange	Dehydration resulted in the lowest GI, while all cooking methods resulted in a moderate GI	[126]
